# Determinants of young people's sexual behaviour concerning HIV and AIDS in the practice population of a university health centre in Lagos, Nigeria

**DOI:** 10.4102/phcfm.v3i1.219

**Published:** 2011-06-10

**Authors:** Olayinka O. Ayankogbe, Kofo Odusote, Mopelola O. Omoegun, Victoria Ofoha, Ayoade Adedokun, Kehinde O. Abiola

**Affiliations:** 1 Department of Community Health and Primary Care, College of Medicine, University of Lagos, Nigeria; 2 Health Center, University of Lagos, Nigeria; 3 Department of Guidance and Counseling, Faculty of Education, University of Lagos, Nigeria; 4 Department of Family Medicine, Lagos State University Teaching Hospital, Ikeja, Nigeria; 5 Department of Laboratory Services, Health Centre, University of Lagos, Nigeria

## Abstract

**Background:**

AIDS has been a scourge of universities in Africa for a long time. This study was launched at ground-level to fight the dreaded disease by concentrating on young people and to counter the ignorance that surrounds the disease even in numerous African universities. This study of the student community was carried out by family doctors at the University Health Department to determine the prevalence of the determinants of young people's reproductive health behaviour.

**Objectives:**

This study is aimed at determining young people's sexual behaviour concerning HIV and AIDS in the practice population of a university in Lagos, Nigeria.

**Method:**

Self-administered 63-item questionnaires were distributed amongst 2000 randomly selected students of the University of Lagos, Lagos, Nigeria in September 2005, using a semi-structured form of the Comprehensive Youth Survey questionnaire, developed by FOCUS (led by Pathfinder International, Futures Group International and Tulane University School of Public Health).

**Results:**

The age distribution of the respondents was designated in the age groups of 15–19 years (15.8%), 20–24 years (60.1%), 25–29 years (19.6%), 30–34 years (2.8%). Demographics of note were that 88.3% of the fathers of the respondents were literate and that 94.5% of the fathers earned more than one US $ per day. The majority of the respondents (99.1%) indicated adherence to one religious faith or the other and 58.8% believed definitely that religion shaped their attitudes about sexual intercourse and sexuality. More than half (64.0%) denied having had sex at all in the three months preceding the study. Furthermore, 68.8% affirmed that it was common amongst friends of their age to use condoms. A significant number of respondents (65.5%) thought that their friends have drunken alcohol. Almost all of the respondents (94.3%) had a positive perception of their family.

**Conclusion:**

The Programming for HIV and AIDS Reduction on university campuses in Africa should be conducted comprehensively rather than monothematically and should, take into consideration the five thematic areas of behaviour change communication amongst young people concerning their reproductive health.

## Introduction

AIDS remains a dreaded disease^1^ in Africa, and every effort should be made to prevent it especially in young people who are disproportionately affected.^2, 3, 4, 5, 6^ In his seminal report on case studies of seven Universities in Africa on HIV and AIDS in 2001, Kelly^7^ concluded that a thick coat of ignorance surrounds the disease in many African universities. This cloak is amply lined with layers of secrecy, silence, denial and fear of stigmatization and discrimination.^7^ Records that name the disease available in University Health Centres are just being upgraded.^7^ Even information on staff and student mortality is just being improved.^7^ However, sexual activity, especially amongst the students, is rampant in all these universities, both in Africa and all over the world.^8, 9, 10^

Several studies on sexual related behaviour of students in the universities also corroborate the extent of high risk behaviour for HIV and AIDS present in these citadels of higher learning, whether in developed or developing countries.^11, 12, 13, 14, 15, 16^

Extensive research into adolescent and young people's behaviour concerning reproductive health has identified five thematic areas that influence these behaviours.^4, 17, 18, 19, 20^ These areas comprise firstly, the individual characteristics of young people, including their knowledge, attitudes, beliefs, values, motivations and experiences. Secondly, their sexual partners and peers and thirdly, their families and the adults in the community play a role. Fourthly, institutions that support the youth and provide opportunities, such as schools, workplaces and religious organisations influence young people's behaviour. Finally communities, through which social expectations are transmitted about gender norms, sexual behaviour, marriage and child bearing, is an important thematic area.^17, 18, 19^

There were no definite figures of reproductive health problems,^7^ but, observations showed that these could be substantial, as the Centre was dealing with a very large student population. Using some of the processes of Community Orientated Primary Care,^21, 22^ a study was conceived to investigate the problems that prevail concerning reproductive health behaviour in the population of the general or family practice.

Behaviour Change Communication regarding HIV and AIDS has been ongoing in the community surrounding the University of Lagos from the first report of HIV or AIDS in Nigeria in 1981. In 2005, activities specifically targeted at the Lagos University students gained momentum. Although undocumented scientifically, these activities included peer education, dissemination of Information Education Communication (IEC) materials, the formation of a student's anti-AIDS club and the preparation of a draft policy document for HIV and AIDS for students and workers on campus. With the award of an intervention grant by PEPFAR (United States Presidential Emergency Programme Fund for Aids Reduction) in September 2005, a baseline study of the student community was carried out by family doctors (family physicians and medical officers). This study was undertaken at the health centre in collaboration with a multidisciplinary team of sociologists, graduate nurses and public health physicians, to determine the prevalence of the determinants of young people's reproductive health behaviour in this practice population of the University Health Centre.

## Ethical considerations

Ethical considerations was obtained from the Lagos University Teaching Hospital Research and Ethics Committee. Written informed consent was obtained from each of the participants.

## Method

The University of Lagos Health Centre is a general practice facility run by generalist doctors and medical officers with Master degrees in Public Health. In 2006, the facility attended to a total of 33 857 patients comprising 5618 student patients (out of a total of 30 000 students), 8027 staff patients and 20 192 dependent patients (i.e. the spouses and children of students and staff).

Self-administered 63-item questionnaires were distributed amongst 2000 randomly selected students from the University of Lagos in Lagos, Nigeria. The university has a total student population of 30 000 (both in campus and off campus). The standard Comprehensive Youth Survey questionnaire, developed by FOCUS (a partnership of Pathfinder International with the Futures Group International and Tulane University School of Public Health), was used. This instrument is an internationally validated instrument used in surveys of reproductive health behaviour in young people all over the world. It covers the five thematic areas of youth behaviour concerning reproductive health. These questionnaires were distributed during official school sessions. A 3-stage multistage sampling design was used to select 200 respondents from each of the 10 faculties of the university including the College of Medicine. In the first stage all the faculties were selected. In the second stage all the departments in each faculty were selected and the 200 respondents from each faculty were distributed according to the number of departments in that particular faculty. Simple random samples were then taken by balloting to select the final number of students from each department. The minimum sample size of 1536, (rounded up to 2000) was arrived at by using the formula:

*(1.96)*^2^*(p)*(*1-p)/d*^2^

where *p* is the prevalent rate (0.5) and

*d* is the margin of acceptable error (2.5%).

Postgraduate student volunteers, trained in data collection through a one-day workshop, distributed the questionnaires. A pilot survey was conducted using a nearby tertiary institution to restructure the survey questionnaire to meet local needs. Data from the questionnaires were fed into the computer and analysed using the software EPIINFO version 6.04b. All bona fide students of the university were expected to participate in the study whether they stayed on campus or off campus.

A total of 1891 questionnaires were retrieved, equalling a response rate of 94.6%.

## Results

The *Age Distribution* (Table 1) shows that the age group of 15– 24 years constituted 60.1% of the study group. The majority (91.1%) were not married, as can be expected from their ages and 88.3% of the fathers were literate (i.e. they have had more than 11 years of continuous education).

**TABLE 1:** The demographic profile of the respondents with reference to their age, gender, relationship status and their home background.

Profile	Frequency	%
**Age group (years)**		
15–19	299	15.8
20–24	1137	60.1
25–29	370	19.6
30–34	53	2.8
35–39	17	0.9
40 and above	15	0.8
**Gender^a^**		
Male	694	36.7
Female	1197	63.3
**Relationship status**		
Single	1724	91.1
Married	134	7.1
Divorced	4	0.2
Separated	4	0.2
Live-in lover	25	1.3
**Religious denomination**		
None	19	1
Roman Catholic	327	17.3
Protestant (Anglican, Methodist, etc.)	96	5
Protestant (Pentecostal)	777	41.1
Moslem	327	17.3
Indigenous (white garment)	97	5.1
Others	248	13.1
**Father's level of education**		
None	81	4.3
Primary school	108	5.7
Secondary school uncompleted (=JSS3)	32	1.7
Secondary school completed (>JSS3)	159	8.4
Post-secondary	255	13.5
University or polytechnic	1256	66.4

**Total**	**1891**	**100**

*Source*: Authors’ original data

a 63.3% were female students whilst 36.7% were male students, giving a female to male student ratio of 1.7/1.

**TABLE 2:** The social class and income of the respondents’ fathers.

Description	Frequency	%
**Father's social class^a^**		
I: Professional occupations (doctors, etc.)	879	46.5
II: White collar workers (shop owner, etc.)	197	10.4
III: Formally trained occupation (police etc.)	76	4
IV: Trained occupations with overalls (welders, etc.)	38	2
V: Untrained occupations – no overalls (guards)	13	0.7
VI: Untrained occupation – overalls (gardeners etc.)	15	0.8
Other (unclassifiable)	673	35.6
**The average income of the father in naira per month (1USD = N100)^b^**		
Less than 2000	59	3.1
2000–3999	40	2.1
4000–5999	79	4.2
6000–7999	76	4
8000 and above	1.638	86.6

**Total**	**1891**	**100**

*Source*: Tropical doctor; Authors’ original data

USD, American dollar; N, naira.

a Social classes are classified according to a Roman numeral numbering system.

b Note that 94.5% of the students’ fathers earn more than 1 US $ per day.

**TABLE 3:** Personal HIV related knowledge and attitude.

Description	Frequency	%
**Involvement with extra- curricular activities on campus (respondents chose more than one option)**		
Yes	-	40.7
No	-	59.3
If yes	-	-
Sport clubs or club teams	232	-
Drama clubs	77	-
Debate clubs	23	-
Academic clubs	107	-
Religious clubs	516	-
Other	54	-
**Meaning of safe sex (respondents chose more than one option)**		
Abstaining from sex	789	-
Using condoms	876	-
Avoiding multiple sexual partners	571	-
Avoiding sex with prostitutes	337	-
Avoiding anal sex	244	-
Others	25	-
**Preferred facility for reproductive health problems (respondents chose more than one option)**		
Clinic or hospital	1475	-
Chemist	36	-
Health worker	97	-
Peer counsellor	38	-
Youth Centre	23	-
Friend	63	-
Parent	149	-
Relative	14	-
Lecturer	20	-
Other	50	-

**Total**	**1891**	**100**

*Source*: Authors’ original data

**TABLE 4:** Knowledge and attitudes that respondents revealed on how to safeguard themselves against AIDS.

Variable	Frequency	%
**What can a person do to avoid AIDS (respondents chose more than one option)?**		
Avoid sex completely	1128	-
Stay faithful to a partner	1136	-
Encourage partner to stay faithful	958	-
Use condom for every act of sexual intercourse	776	-
Avoid sharing needle	954	-
Avoid commercial sex workers	821	-
Avoid casual sex	838	-
Avoid circumcision in unauthorized places	830	-
Other (to enumerate in footnotes if possible)	-	-

**Total**	**1891**	**100**

*Source*: Authors’ original data

In general, respondents had a more than average knowledge about HIV and AIDS and the prevention of it and were aware of where to find help in a majority of cases (Table 3).

### Religion

A significant percentage (58.8%) has a firm conviction that religion shapes their attitudes about sexual intercourse and sexuality whilst 24.6% have a firm belief that it does and 5.4% accept that it does.

The pertinent religious debate about AIDS and sin in relation to this study reveals that 90.5% of the respondents do not believe that people who contracted HIV or AIDS are sinners.

### Behavioural practices

When asked how many times they had sexual intercourse in the three months preceding the study, 64% denied having had sex at all. Of the rest, 6.4% admitted to have had sex once, and 6.7% admitted to two to three times. Furthermore, 3.1% admitted to four to five times, 3.2% to six to eight times and 5.4% to nine or more times, whilst 11.1% said that they could not remember. That means that 24.8% admitted to have had sexual intercourse in the three months preceding the study.

In the evaluation of the number of partners they have had sex with in the three months preceding the study, 63.1% said none, 25.2% said one, 5% said two, 2.4% said three whilst the rest had partners varying from four to eight (2.1%). Consequently a total of 9.5% admitted to having had multiple sexual partners.

The social aspect of coerced sexual intercourse revealed that 20% of the students had been forced to have sexual intercourse, whilst 78% had not been forced (Table 5). The results also showed that 87.6% of the students had never received anything in exchange for sex whilst 9.1% had and 26.2% are currently in a same sex relationship.

**TABLE 5:** An assessment of the self-efficacy of the respondents.

Assessment specifications	Definitely could not		Probably could not		Probably could		Definitely could		Not sure		Total
					
	*n*	%	*n*	%	*n*	%	*n*	%	*n*	%	*n*
**If you do not want to have sex, how confident are you that you would be able to refuse sexual intercourse?**											
With a person you have known for 3 months	547	28.9	176	9.3	212	11.2	737	39	219	11.6	-
With a person who offers you gifts	787	41.6	146	7.7	163	8.6	737	39	209	11.9	-
With a person who has power over you	858	45.4	142	7.5	161	8.5	545	28.8	185	9.8	-
**How confident are you that you will be able to …?**											
Have a sexual relationship with only one person for 6 months	276	14.6	98	5.2	214	11.3	1097	57.7	212	11.2	-
Choose with whom to have sex	233	12.3	76	4	229	12.1	1164	61.6	189	10	-
Avoid sex anytime you do not want	170	9	91	4.8	312	16.5	1176	62.2	142	7.5	-

**Total**	**-**	**-**	**-**	**-**	**-**	**-**	**-**	**-**	**-**	**-**	**1891**

*Source*: Authors’ original data

### Condom use

During the study 68.8% of the respondents affirmed that amongst friends of their age, it was common to use condoms and 47.2% of the students admitted to using condoms.

In the two major areas of assessment of self-efficacy amongst these respondents, the scores were very high (Table 5).

### Mass media influences

The influence of the mass media, such as radio and television, can be seen globally. In this study, the record of the frequency with which students listen to the radio revealed that 65.8% of the students listened to the radio every day or almost every day, 21.8% listened to it at least once per week and 3.8% listened at least once per month. Very few did not listen to the radio regularly; 2.9% listened to it less than once per month and a very low percentage of 2.5% of the students never listened to the radio.

When asked how many times they had listened to Radio Unilag, in the preceding two weeks to the study, only 39% said zero number of times. The rest has listened to it in varying numbers of times ranging from once to 60 times.

The most significant data on peer and partner pressure (Table 6) are that 57.9% did not agree that they should put pressure on their partner to have sex with them; unfortunately, less, but still a significant portion, that is, 48.6% affirmed that unmarried female students in the university community encourage other girls to have sex with male students or older men. At the same time, 49.6% affirmed that unmarried male students in the university community encourage other male students to have sex with female students or older women. It is encouraging that 60.5% said that there was no pressure from their friends for them to have sexual intercourse.

**TABLE 6:** Peer and partner pressure.

Variable	*n*	%
**Putting pressure on partner to have sex**.		
Agree	465	24.6
Disagree	1097	58
Do not know	329	17.4
**Unmarried female students encouraging other girls to have sex with male students or older men**.		
Yes	919	48.6
No	327	17.3
Do not know	645	34.1
**Unmarried male students encouraging other male students to have sex with female students or older women**.		
Yes	938	49.6
No	302	16
Do not know	651	34.4
**Have you ever been encouraged?**		
Yes	520	27.5
No	1237	65.4
Do not know	134	7.1
**Support amongst friends to wait until marriage before having sexual intercourse**.		
No support at all	397	21
A little support	499	26.4
Moderate support	341	18
Substantial support	654	34.6
**Pressure from friends to have sexual intercourse**.		
No pressure at all	1145	60.5
A little pressure	422	22.3
Moderate pressure	125	6.6
Substantial pressure	199	10.6

**Total**	**1891**	**100**

*Source*: Authors’ original data

Number of respondents is given as *n*.

When the respondents were asked if a peer educator has ever talked to them, 54.6% replied in the affirmative. Of those who have talked to a peer educator, 63.7% would like to talk to one again and 16.4% were undecided.

A significant number of the respondents, 65.5% of the study group, thought that their friends have drunk alcohol and only 19.3% of the respondents thought that none of their friends have drunk alcohol (Table 7). It is encouraging that there was a strong perception amongst the respondents that their friends did not use drugs, that is, 78.4% of the respondents. Smoking seemed to be more common, as 56.9% agreed that their friends have smoked.

**TABLE 7:** The use of substances and the perception of the use of substances by friends of the respondents.

Substance	None of them		A few of them		About half of them		Most of them		All of them		Do not know		Total
						
	*n*	%	*n*	%	*n*	%	*n*	%	*n*	%	*n*	*%*	*n*
Number of friends that have drank alcohol.	365	19.3	785	41.5	91	4.8	272	14.4	85	4.5	293	15.5	-
Friends that have taken drugs	1483	78.4	280	14.8	15	8	59	3.1	40	2.1	15	8	-
Number of friends who have smoked	756	40	921	48.7	74	3.9	108	5.7	28	1.5	4	0.2	-

**Total**	**-**	**-**	**-**	**-**	**-**	**-**	**-**	**-**	**-**	**-**	**-**	**-**	**1891**

*Source*: Authors’ original data

Number of respondents is given as *n*.

### Family influence

Approximately half (52.2%) of the students described their family to be sweet and warm in general. A majority of the respondents (79.8%) said that their parents supported their decisions and 61.9% of this majority said that their parents mostly supported them whilst 17.9% said that they were fully supportive (Table 8). A smaller percentage (10.5%) revealed that they did not know whether their parents supported their decisions.

**TABLE 8:** The influence of family relationships in the respondents’ lives.

Variable	Frequency	%
**Description of Family in General**.		
Sweet and warm	987	52.2
Good	645	34.1
Fair	146	7.7
Not very good but tolerable	64	3.4
Intolerable and would like to leave	21	1.1
Do not know	28	1.5
**Parental support for personal decisions**.		
Not at all	83	4.4
No	102	5.4
Yes	1169	61.8
Yes very much	338	17.9
Do not know	199	10.5
**Having a good relationship with the brothers and sisters**.		
All the time	857	45.3
Usually	620	32.8
Sometimes	323	17.1
Not usually	61	3.2
Never	15	0.8
Do not know	15	0.8

**Total**	**1891**	**100**

*Source:* Authors’ original data

Most of the students (78.1%) had a good relationship with their brothers and sisters, and 45.3% of those students had a good relationship with their brothers and sisters all the time, whilst 32.8% usually related to one another. The rest of them, that is, 17.1%, sometimes related to one another (Table 8).

## Discussion

This study set out to explore the distribution of known determinants that influence behaviour change amongst the youth concerning HIV and AIDS. The five thematic areas that influence behaviour change are strong in this West African citadel of learning. These thematic areas were not explored in this particular study, but the implications are enormous for the control of HIV and AIDS on campuses not only in Nigeria, but across the continent.

### Demographics

The socioeconomic milieu that puts people at risk of HIV and AIDS is absent in this student population. With the majority of the students in Social Classes I and II^23^ as classified in Table 2, and income brackets of N8000 per month and above, they are not in the poverty trap, nor are they in the low socioeconomic group that is associated with high HIV and AIDS prevalence. Students in a higher educational institution have a tremendous advantage, for^,^ higher education students are more likely to have sexual education^25, 26^. Furthermore, condom use in particular has been found to increase in student populations with higher levels of education.^27^

### Personal HIV related knowledge and attitudes

Personal HIV related knowledge is as high in this student population as in other university student populations in Africa^26^; this can also be said about most universities in Nigeria. However, unlike most other studies where permissive attitudes prevail, attitude levels towards sexual activity are low.^20, 28, 30^ This suggests that behaviour change communication strategies finally seem to be working in this student community in Lagos judging from the fact that peer education, behaviour change communication and HIV mitigating policy processes were on-going prior to the study.

The perception of risk of HIV infection is low. This is the general trend amongst Nigerians of all ages and may be attributed to the fact that Nigeria has a low prevalence rate of 4.8%.^54^ This low rate of perception of risk follows findings in the developed world too.^28, 29^ It is universally low amongst young people, who are eternal optimists. This underscores the fact that preventive efforts concerning HIV and AIDS must become a lifestyle.

### Religion

In this series, religious influence was significant in influencing attitudes to sex and consequently preventative behaviour towards HIV and AIDS. Although not deeply explored in this student population, high levels of religiosity are associated with low levels of sexual activity. Few studies are available to compare with those in Nigeria. Nevertheless, religion plays a major role in the consciousness of Nigerians. Many HIV and AIDS prevention activities are now handled successfully by faith-based organisations, a testimony to this trend. This trend is also found in the rest of Africa.^20, 27^ This seems to be unique to Africa and in particular to Nigeria.^25^

### Behavioural practices

Rates of sexual activity amongst students in this series are consistently low at 24.8%, although when compared to secondary school students in Malaysia, another developing country, the rates of sexual activity in this study are higher (12.6% in Malaysia).^31^ Much higher rates are found in developed countries.^33, 34, 35^ The incidence of multiple sexual partners is low as well. The only explanation is that the five thematic areas for young people's preventive behaviour concerning HIV and AIDS are very strong in this student population.

In a study that was carried out in Sweden amongst the general population, sexual activity remains high amongst young people aged 16–25 years.^32^ High rates of sexual activity were expected in this study, as Lagos is perceived to be a cosmopolitan and sexually notorious city in Nigeria, even by Nigerians.

It is pertinent to note that adolescent African students remain more sexually active than their college colleagues.^30^

### Condom use

Condom use is moderate amongst this study series (47.2%). This is quite unusual as condom use is generally low amongst young people all over the world.^35, 32, 4, 38^ In Nigeria, however, high condom use is more probable and driven by fear of pregnancy rather than any other reason because pregnancy in a female student generally signals the end of her formal education. The high level of use amongst study participants may be because of enhanced negotiation skills.^36^

### Self-efficacy

Self-efficacy concerning the ability to engage in risky sexual behaviour was very high amongst study participants. Although this is a self-reported activity, this is commendable. Amongst Nigerian youths, there is a very high degree of self-confidence in almost everything they do. Whether this confidence is real or imagined is another matter entirely

This high level of self-efficacy pervades in the attitudes of college students on the African continent too.^26^ Studies consistently show that self-efficacy translates into safer sex practices generally.^40, 41^ However, high self-efficacy does not always translate into safe sexual behaviour amongst college students.^34^

### Mass media influences (radio)

One good development amongst young people is their love for music and the radio and other electronic media. The majority of the university students in this study listen to the radio every day and especially to Radio Unilag. Young people in Nigeria are fanatical football fans and the radio is the cheapest and fastest means of gathering news about their favourite teams, whether in Nigeria or in Europe. Exposure to mass media-related HIV and AIDS programming has been linked to attitudinal and behavioural changes both in Nigeria and in the rest of the world.^42^ In a study conducted in China, exposure to multiple sources of HIV information (where at least one source was the mass media) was significantly related to HIV knowledge and a less stigmatizing attitude towards People Living With HIV and AIDS (PLWHA).^42^ Mass media in China has been a major source of HIV information to the public.^42^ Enhancing the content and penetration of HIV and AIDS campaigns within various channels of the media can be an important strategy in disseminating HIV knowledge and reducing HIV-related discrimination ^42^ The possibility of reaching millions of young people through global networks with minimal marginal costs after production creates a new paradigm for reaching an important segment of young people.^43, 44^

### Substance use and the perception of substance use

Crack cocaine smoking has been identified as a risk factor for HIV and AIDS;^45, 46, 5^ however, as little as 20.8% of the students in the study group are suspected of taking hard drugs (Table 7) The substance most commonly abused by young people is tobacco, either as cigarettes or as snuff.^49^ This was also the case in this study, with rates being as high as 60%. The risk, however, of contracting HIV and AIDS is more readily associated with hard drug use than with cigarette smoking.^46, 47, 49^ The other substance referred to in this study was alcohol. Increased sexual activity coupled with alcohol abuse increases the risk of contracting HIV and AIDS.^50^ Quite a high proportion of students in this study drank alcohol (approximately 64%, Table 7). Further studies are needed to identify the drugs used by the 20.8% who were classified as hard drug users.

### Peer and partner pressure

On average, the pressure exerted by these students on each other to perform adverse sexual acts was not high. There is no obvious explanation for this. This was in contrast to other studies on the continent where peer pressure amongst university students to have sex is high.^20, 51, 50^ The question is whether this lower peer pressure was a result of the high religiosity of these students. There is no doubt that previous HIV and AIDS programming in this student community has influenced the trend towards the high prevalence of these positive determinants.

### Family influence

Family influences amongst the respondents of this study are very strong even for nonreproductive issues. Family ties in Nigeria remain very strong which may have influenced HIV and AIDS behaviour in this student population, although it was not explored in this study.

## Conclusion

Programming for HIV and AIDS reduction on university campuses in Africa should be comprehensive rather than vertical single intervention based. In doing so, the five thematic areas of behaviour change communication amongst young people concerning reproductive health, backed by a health centre run by generalist doctors practicing Community Oriented Primary Care, are a sine qua non.

### Research significance

Most programming and evaluation of interventions in sexual behavior amongst young people has been monothematic and population based. We present a multithematic programming that emanates from a family practice facility; an example of a partial process of Community Oriented Primary Care (COPC).

### Limitations of the study

The usual reservation about the absolute reliability of self- reported observations in a research study is made. However the sheer number of respondents puts this reservation at the most minimum level.
